# Functional training improves cognitive function, functional fitness, and BDNF levels in older women with mild cognitive impairment: a randomized controlled trial

**DOI:** 10.3389/fphys.2025.1638590

**Published:** 2025-09-11

**Authors:** Salviano Resende-Silva, Antônio Gomes de Resende-Neto, Alan Bruno Silva Vasconcelos, Marcos Raphael Pereira-Monteiro, Alan Pantoja-Cardoso, Laíza Ellen Santana Santos, Newton Benites Carvalho Lima, José Lucas de Jesus Santos, José Carlos Aragão-Santos, Dulce Marta Schimieguel, José Ronaldo dos Santos, Marzo Edir Da Silva-Grigoletto

**Affiliations:** ^1^ Postgraduate Program in Physiological Sciences, Federal University of Sergipe, SãoCristóvão, Brazil; ^2^ Postgraduate Program in Health Sciences, Federal University of Sergipe, São Cristóvão, Brazil; ^3^ Department of Health Education, Federal University of Sergipe, Lagarto, Sergipe, Brazil; ^4^ Department of Physical Education, State University of Pará, Altarmira, Pará, Brazil; ^5^ Postgraduate Program in Pharmaceutical Sciences, Federal University of Sergipe, São Cristóvão, Brazil; ^6^ Graduate Program in Physical Education, Physical Education Department, Santa Cruz State University, Ilhéus, Bahia, Brazil; ^7^ Physical Education Department, Federal University of Sergipe, São Cristóvão, Sergipe, Brazil; ^8^ Graduate Program in Physiological Sciences/Physical Education Department, Federal University of Sergipe, São Cristóvão, Sergipe, Brazil; ^9^ Graduate Program in Health Sciences, Physical Education Department, Federal University of Sergipe, São Cristóvão, Sergipe, Brazil; ^10^ Department of bioscience, Federal University of Sergipe, Itabaiana, Sergipe, Brazil; ^11^ Department of Physical Education, Federal University of Sergipe, São Cristóvão, Brazil

**Keywords:** ageing, exercise, health, functional status, personal autonomy

## Abstract

**Introduction:**

To oppose the aging-related cognitive and functional decline, the efficacy of different training methods has been tested, aiming body multisystemic adaptations. In this sense, Functional Training (FT) has shown relevant results in the physical fitness of older adults. However, little is known about its impact on older women with mild cognitive impairment (MCI).

**Objective:**

To compare the effects of functional and aerobic training on cognitive function and functional fitness in older women with mild cognitive impairment.

**Methods:**

Sixty-eight older women completed a 16-week intervention through three groups: 1) Functional Training (FT: n = 28; mean age = 67.5 ± 4.8 years), 2) Aerobic Training (AT: n = 22; mean age = 66.3 ± 4.6 years), and 3) Control Group (CG: n = 18; mean age = 67.5 ± 4.6 years). The training sessions did occur three times per week, with a duration of 50 min per session for both exercised groups. The training load was quantified daily in the FT group. Cognitive function, serum levels of Brain-Derived Neurotrophic Factor (BDNF), and functional fitness were assessed pre- and post-intervention.

**Results:**

FT and AT groups showed improvements in cognitive status (FT: d = 0.99, p ≤ 0.001; AT: d = 0.97, p ≤ 0.001) and semantic memory (FT: d = 0.95, p < 0.001; AT: d = 0.97, p < 0.001); however, only FT led to improvements in executive function (d = 0.63, p = 0.043) and increased serum BDNF levels (d = 0.95, p = 0.011). Regarding functional fitness, both groups showed improvements in gait and stand up ability, as well as cardiorespiratory fitness. However, only FT improved dexterity and upper limb strength. No statistically significant differences were observed between the FT and AT groups in the measured outcomes.

**Conclusion:**

Both FT and AT improve cognitive function and functional fitness in older women with mild cognitive impairment. However, in addition to eliciting a greater magnitude of effects on some outcomes, FT was the only intervention to modulate peripheral BDNF concentration.

## 1 Introduction

Mild Cognitive Impairment (MCI) is an intermediate state between normal cognitive aging and dementia, characterized by changes that do not significantly affect daily activities. In turn, it hinders the cognitive functions involved in the acquisition, processing, storage, and use of information, with an annual conversion to dementia of approximately 15% (Gure et al., 2013). This cognitive decline is associated with a reduction in brain density and volume of approximately 2.82% over a 6-month period (Teixeira et al., 2018), which impairs the connectivity and expression of neurotrophins involved in neuroplasticity, such as Brain-Derived Neurotrophic Factor (BDNF) (Leal et al., 2014; Li et al., 2021), compromising cognitive function (Tolea et al., 2015; Zahodne et al., 2013). This condition is prevalent in older women due to their longer life expectancy and the reduction in sex hormone production during the post-menopausal period (Hägg and Jylhävä, 2021). Furthermore, this decline can be accelerated by factors such as arterial hypertension, obesity, and physical inactivity (Erickson et al., 2022; Smid et al., 2022).

Physical exercise stands as the foremost non-pharmacological strategy to counteract physical inactivity (Erickson et al., 2022; Van Dyck et al., 2023). Exposure to aerobic exercise enhances cardiorespiratory capacity and cerebral vascularization (Huang et al., 2024), thereby increasing the supply of oxygen and nutrients to neural cells (Crichton et al., 2014). This, in turn, leads to greater cerebral connectivity (Holzschneider et al., 2012), improving learning (Voss et al., 2024) and decelerating cognitive decline (Brinke et al., 2015; Norling et al., 2024). In parallel, neuromuscular stimuli continuously activate areas involved in motor planning and execution (Lee et al., 2024), consequently impacting cerebral connectivity (Grégoire et al., 2019; Hewston et al., 2021). Both types of stimuli promote the release of myokines, interleukins, and neurotrophins, fostering positive adaptations from exercise practice (Islam et al., 2021; Ferrer-Uris et al., 2022; Andronie-Cioara et al., 2023; Herold et al., 2019; Azevedo et al., 2023; Duarte Martins et al., 2024; Vints et al., 2024). The integration of physical and motor components demonstrably improves global cognition, language, executive function, attention, and concentration (Huang et al., 2022; Rondão et al., 2022; Suzuki et al., 2012; Vaughan et al., 2014; Zhu et al., 2018). In healthy older adults, a positive association exists between physical activity levels and cognitive function, alongside enhanced task performance. These benefits can be further amplified in older adults with MCI (Xu et al., 2024; Wang et al., 2024).

Among various modalities, functional training (FT) is a multicomponent, multiplanar, and multiarticular approach that emphasizes activities of daily living and leverages the principle of specificity (Silva-Grigoletto et al., 2020). This is achieved by applying stimuli that develop muscular strength and power in basic functional patterns, such as pushing, pulling, carrying, and squatting. Additionally, the session incorporates acceleration and deceleration exercises, typically organized into blocks (Silva-Grigoletto et al., 2020; Antonio Gomes De and Marzo). FT demonstrates significant effects on physical fitness, with effect sizes ranging from moderate to large (Rocha et al., 2023; Aragão-Santos et al., 2020). However, its impacts on the cognitive function of older adults with MCI are not yet fully elucidated (Pantoja-Cardoso et al., 2023). Prospective studies suggest incorporating motor complexity and dose control into FT protocols as an alternative to induce cognitive adaptations (Da Silva-Grigoletto et al., 2024).

On the other hand, Aerobic Training (AT) is considered an effective intervention in the prevention and treatment of cognitive impairments by stimulating the release of myokines and increasing cerebral blood flow. Different types of AT demonstrate promising effects on cognition in older adults with MCI, such as continuous walking (Khan et al., 2023; Nocera et al., 2017), high-intensity interval training (HIIT) (Mekari et al., 2020)), and rhythmic training (Zhu et al., 2022). However, these protocols show a smaller magnitude of effect on the physical fitness of older adults when compared to neuromuscular training methods (Gure et al., 2013; Altieri et al., 2021). Furthermore, it is believed that appropriate levels of muscle strength and power are associated with better overall cognitive performance and in its subdomains (Mavros et al., 2017).

Therefore, our study compared the effects of functional and aerobic training on cognitive function, serum BDNF levels, and functional fitness in older women with mild cognitive impairment. Additionally, the present intervention proposal has novel characteristics not yet evidenced and a precise quantification of the training load throughout the intervention period. Our initial hypothesis was that FT is as effective as AT to improve the cognitive function; however, it provides greater effects on functional fitness and in increasing peripheral BDNF levels.

## 2 Materials and methods

### 2.1 Study design

**FIGURE 1:**
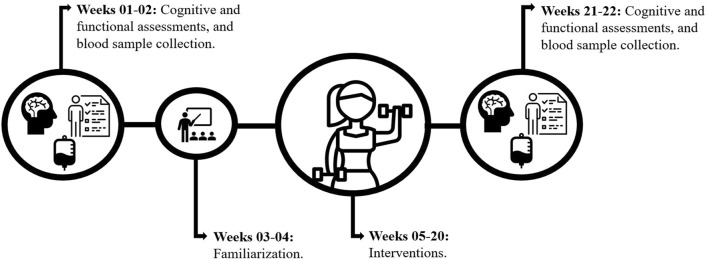
Experimental design.

This was a 22-week randomized controlled trial. Physical and cognitive assessments were conducted over a 4-week period: 2 weeks before the intervention and 2 weeks after the intervention, to collect pre- and post-intervention data. Additionally, 2 weeks were dedicated to familiarizing the participants with the exercises, and 16 weeks were allocated for the implementation of the training protocols (Figure 1).

### 2.2 Participants

**FIGURE 2:**
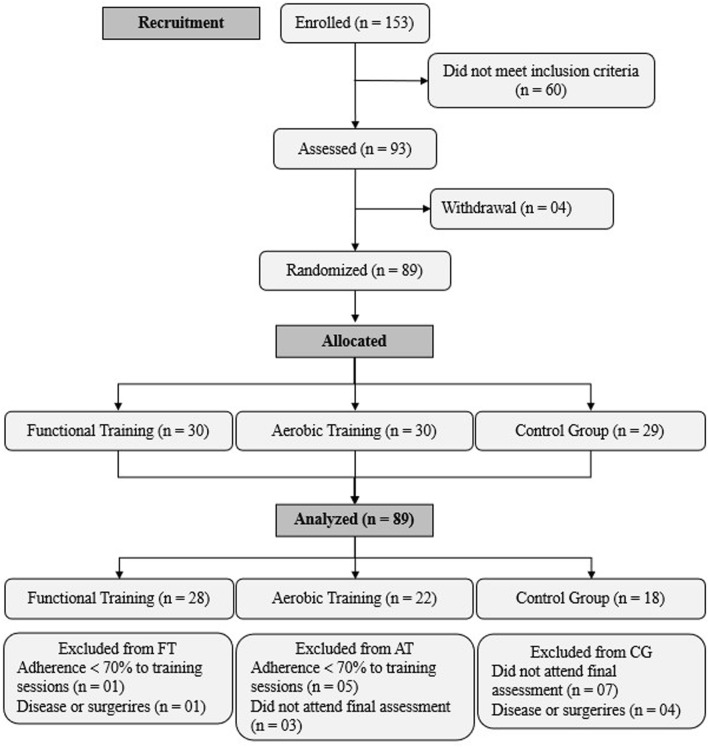
Schematic representation of the screening, allocation, and intervention processes of the study.

The participants were recruited from the community using leaflets and flyers distributed around the Federal University of Sergipe, through a non-probability sampling method (Figure 2). To detect between-group differences with an alpha level of 0.05 and assuming a large effect size, we would need a total of 45 participants in our study to achieve a power of 0.80. Notably, we considered a 20% sample loss during the intervention, totaling 54 participants. We randomized the participants based on the residual value of the means of all performed tests, which were organized in ascending order for every three values using Microsoft Excel software.

The selection process for participants followed several inclusion criteria: 1) female sex; 2) physically independence; 3) being literate; 4) not involved in any hormone replacement therapy; 5) scoring >8 and <26 points on the Montreal Cognitive Assessment (MoCA) (Malek-Ah et al., 2024); 6) reporting complaints of memory problems (Gifford et al., 2014); and 7) without diagnosed neurological diseases (dementia, Parkinson’s disease, Alzheimer’s disease, multiple sclerosis, and depression), recent surgeries, vision, hearing, musculoskeletal, and cardiorespiratory problems that would prevent the practice of high-intensity physical exercise. Participants with an attendance rate below 70% in the training sessions and those who did not attend the final assessment were excluded from the study analyses.

After the application of the eligibility criteria, the participants signed the Free and Informed Consent Form. This study was conducted in accordance with the Declaration of Helsinki and approved by the institution’s Research Ethics Committee (CAAE: 76026223.4.0000.5546; No. 6,598,301). Furthermore, it is registered in the Brazilian Registry of Clinical Trials under protocol RBR-89svjw3 (available at https://ensaiosclinicos.gov.br/rg/RBR-89svjw3).

### 2.3 Physical training protocols

Three weekly sessions were conducted, each lasting approximately 50 min, over a period of 16 weeks, totaling 48 trainings sessions, with a minimum interval of 48 h between sessions. Both training protocols were supervised by experienced physical training professionals for older adults. There was one professional for every five participants to ensure safety and correct exercise execution.

#### 2.3.1 Functional training

The sessions were composed of three parts: 1) preparation for movement with joint mobility exercises (glenohumeral, thoracic, hip, and ankle) and muscle activation (static marching and squats); 2) stimuli directed at speed, coordination, agility, and muscle power; and 3) exercises focused on muscle strength in basic functional patterns of pushing, pulling, carrying, and squatting, which reflect activities of daily living. Both blocks, 2 and 3, were performed in a circuit format (Table 1).

**TABLE 1:** Description of the session structure and progressions implemented during the 16 weeks of Functional Training.

	Session structure		
Mobility and activation	(Cervical) – Frontal flexion and extension, abduction and adduction		
	(Glenohumeral) – Horizontal flexion, extension and shoulder flexion, rotation		
	(Thorax/Hip) – Shoulder protraction and retraction, unilateral thorax abduction; Trunk flexion, alternating leg raises, hip circumduction		
	(Knee/Ankle) – Forward step, dorsiflexion and plantarflexion, squat		
	(Coordination) – Knee raises with contralateral hand touches, adding counting rhythm and pause		
	(Muscle and Cognitive Activation) – Simple multidirectional movement patterns with term correlation		
Total time	10 min total, 01 set with 10 repetitions per exercise		

Exercise progression			
Sessions	01–16	17–32	33–48
Neuromuscular 01 (Circuit Format)	Parallel stance and straight throw against the wall	Parallel stance and throw to the ground	Parallel stance and upward throw with jump
	Forward jump	Jump with diagonal entry	Lateral jump (gallop)
	Forward entry with one foot in each square at a time	Forward stepping with lateral displacement	Forward entry and bilateral exit with forward jumps (Hopscotch)
	Forward displacement with cone touch	Lateral displacement with change of direction touching the cone with a jump	Lateral and forward displacement with jumps over cones
	Vertical waves with the battle rope	Horizontal waves with the battle rope	Alternating vertical waves with the battle rope
	5-m forward sprint	Bipedal jump with 10-m sprint	Asymmetrical foot jumps with 10-m sprint
Total time	15 min total, 06 exercises, 2 rounds, 75 s per station, work-to-rest ratio 1/2		
Neuromuscular 02 (circuit format)	Front Squat (Load)	Front Squat (Load)	Front Squat (Load)
	Neutral Grip Suspension Trainer Row	Alternating Grip (Neutral and Supinated) Row	Alternating Grip (Pronated and Supinated) Row
	Bilateral Hip Thrust	Bilateral Hip Thrust with Simultaneous Bilateral Push-Off	Unilateral Hip Thrust
	Pull-up	Pull-up with Alternating Knee Raise on the Eccentric Phase	Pull-up with Leg Extension and Ipsilateral Touch on the Supporting Leg
	Bilateral Deadlift (Load)	Bilateral Deadlift (Load)	Bilateral Deadlift (Load)
	*Bilateral Farmer’s Walk*	*Bilateral Loaded Farmer’s Walk in Zig-Zag Pattern*	*Unilateral Loaded Farmer’s Walk in Zig-Zag Pattern*
	Supinated Grip Resistance Band Pull-down	Bilateral Pull-down with Squat on the Eccentric Phase	Bilateral Pull-down with Contralateral Leg Flexion
Total time	25 min total, 07 exercises, 2 rounds of 8 to 12 repetitions, 100 s per station, work-to-rest ratio 2/1		

Exercises complexity progressions occurred every 16 sessions. In the second part, this was achieved through variations in planes (sagittal, frontal, and transverse) and direction of movement (vertical, horizontal, and diagonal). In the third part, progression explored motor complexity by altering the number of segments and planes, with dual motor tasks associated with increments in movement speed (La Scala Teixeira et al., 2019; Saeterbakken et al., 2011). The participants underwent supervised and progressive training, with daily quantification of the load (Volume load = sets x repetitions x load) for the eight exercises of the neuromuscular part (Buskard et al., 2018; Genner and Weston, 2014).

#### 2.3.2 Aerobic training

The applied systematization possesses particularities not yet evidenced in scientific literature, such as the combination of three training methods within the same session, aiming for improvements in agility, balance, coordination, speed, and cardiorespiratory fitness. Thus, the session was divided into four parts: 1) preparation for movement with joint mobility exercises (glenohumeral, thoracic, hip, and ankle) and muscle activation (static marching and squats); 2) continuous running (50% and 70% of heart rate reserve; HRR); 3) rhythmic movements; and 4) high-intensity interval running (Delgado-Floody et al., 2015).

There were no progressions during the weeks; however, the intensity of the session was prescribed between 50% and 80% of HRR, being monitored throughout the session, allowing for the tracking of the average HRR behavior during the parts of the sessions (Karvonen et al., 1957) (Table 2).

**TABLE 2:** Description of the Aerobic Training session.

Session structure	Exercises		
Mobility And Activation	(Cervical) – Frontal flexion and extension, abduction and adduction		
	(Glenohumeral) – Horizontal flexion, extension and shoulder flexion, rotation		
	(Thorax/Hip) – Shoulder protraction and retraction, unilateral thorax abduction; Trunk flexion, alternating leg raises, hip circumduction		
	(Knee/Ankle) – Forward step, dorsiflexion and plantarflexion, squat		
	(Coordination) – Knee raises with contralateral hand touches, adding counting rhythm and pause		
	(Muscle and Cognitive Activation) – Simple multidirectional movement patterns with term correlation		
Total time	10 min total, 01 set with 10 repetitions per exercise		
Continuous part	Continuous running in a corridor		
Total time	10 min total, 70-m course (70m ±20 s)		
Movements performed			
Rhythmic part	Backward step	Forward step	Lateral step
	Backward kick	Forward kick	Lateral kick
	Knee flexion		
	Leg curl		
	Arm flexion	Leg flexion	
Total time	10 min, 10 sets of 8 repetitions per movement		
Interval part	30 Sprints – 12 m		
Total time	10 min, 12 m (01 sprint ±03 s), work-to-rest ratio 1/4		
130, 140 and 150 - Beats Per Minute (Music) (Rhythmic Part)			
05 to 08 – Rating of Perceived Exertion (Session)			
50%–70% Heart Rate Reserve (Session)			

#### 2.3.3 Control group

The control group performed static and dynamic stretching exercises. The exercises began with the muscles of the neck and progressed down to the dorsum of the feet. The session duration was approximately 40 min, with 10 min dedicated to preparation for movement with joint mobility exercises (glenohumeral, thoracic, hip, and ankle) and 30 min devoted to the practice of submaximal stretches, respecting the comfort levels of the participants.

### 2.4 Data collection procedures

Initially, the participants underwent an eligibility assessment, and information such as age, body weight, height, address, motivation, medication use, and chronic diseases was recorded. For the cognitive and physical tests, all measurements were performed by trained and experienced professionals in the data collection procedures, which always occurred in the morning period. The assessors were blinded to the physical training protocols previously performed by the participants.

#### 2.4.1 Categorization instruments

The participants were functionally classified based on the Montreal Cognitive Assessment (MoCA) questionnaire (Nasreddine et al., 2005), the Geriatric Depression Scale and Anxiety Scale (GDS-15) (Yesavage et al., 1982), and the Lawton-Brody Instrumental Activities of Daily Living Scale (Lawton, 1969).

#### 2.4.2 Cognitive assessment

The tests were administered at pre- and post-intervention time points in a closed room, free from visual or auditory distractions, with controlled temperature (23 °C ± 2 °C), and with a single investigator in the room to provide instructions and evaluate the participants. The application of the cognitive tests lasted approximately 30 min, following a pre-established order of administration. To assess the impact of the intervention on cognitive function, the following cognitive tests were administered at pre- and post-intervention time points:

The Montreal Cognitive Assessment (MoCA), with a maximum score of 30 points, was used to evaluate overall cognitive status. The MoCA was also used for screening mild cognitive impairment (MCI), demonstrating a high intraclass correlation coefficient (ICC = 0.88). Reference values for classifying cognitive impairment are scores above 8 points and below 26 points, with a sensitivity exceeding 80% (Malek-Ah et al., 2024; Langa and Levine, 2014).

Animal category verbal fluency was employed to assess semantic memory, language, and executive function. This test is widely used in neuropsychological assessments due to its recognized sensitivity for screening MCI and dementia (Brucki et al., 1997; Mirandez et al., 2017). The verbal fluency test is validated for the Brazilian population, exhibiting moderate to high intraclass correlation coefficients (ICC >0.8) and a sensitivity greater than 79% (Paulsen et al., 1995; Radanovic et al., 2009).

Finally, the Digit Span Forward (DSF) test was administered to assess short-term memory. In contrast, the Digit Span Backward (DSB) test evaluated working memory, where participants had to repeat numerical sequences in reverse order (Schroeder et al., 2012). Both versions demonstrated a high intraclass correlation coefficient (ICC = 0.88) and a sensitivity above 70% (Dell’Oro de Oliveira et al., 2014). These tests have proven efficacy in detecting declines in short-term memory and working memory, respectively (Yoshimura et al., 2023).

#### 2.4.3 Functional fitness assessment

The tests comprising the functional fitness assessment were performed in a randomized order on the same day for all participants. Each test included a familiarization trial followed by three recorded attempts, with a 2-min rest interval between attempts. The investigators provided verbal encouragement during the execution of the tests and additional explanations when necessary. Participants were instructed to perform all tests with maximum effort to select the best value among the three attempts. The tests comprising the functional fitness assessment were time-based and recorded using a digital stopwatch.

##### 2.4.3.1 Stand up ability

Two tests were used to assess chair-rise ability: 1) Five Times Sit-to-Stand Test (FSTS), which indirectly evaluated lower limb power, with participants sitting down and standing up from a 45 cm high chair for five consecutive repetitions (Teo et al., 2013); 2) Floor Rise Test (FRT), which evaluated overall functionality with high demand on core muscles, with the participant rising from a prone position to a standing position without swaying (Santana et al., 2021).

##### 2.4.3.2 Gait ability

Two tests were used to assess gait ability: 1) Timed Up and Go (TUG), which evaluated agility and dynamic balance, where the participant stood up from a chair, walked 3 m, turned around a cone, and returned to the seat (Podsiadlo and Richardson, 1991); 2) 10-m Walk Test (10MWT), which evaluated gait speed, with the participant walking 10 m of a 14-m course, excluding the acceleration and deceleration areas (Lusardi et al., 2003).

##### 2.4.3.3 Dexterity ability

Two tests were used to assess dexterity ability: 1) Gallon Jug Shelf Transfer (GJST), which evaluated upper limb strength and coordination by transferring five gallons of 3.9 kg between a lower and an upper shelf, transferring 1 gallon at a time (Signorile et al., 2007); and 2) Dressing on and taking off a t-shirt (DTTS), which evaluated upper limb mobility and coordination, with the participant donning and doffing a shirt as quickly as possible (Vale et al., 2006).

##### 2.4.3.4 Upper limb strength

Handgrip strength (HGS), which assessed the muscular strength of the upper limbs, was measured using a handgrip dynamometer (Jamar Hydraulic Hand Dynamometer; Homecraft Ltd., ghamshire, United Kingdom), in conjunction with the functional fitness tests (Wang and Chen, 2010). The participant was seated in a chair and applied progressive maximal force with the dominant arm flexed at 90°. The highest value in kilogram-force (kgf) was selected for analysis.

##### 2.4.3.5 Cardiorespiratory capacity

The 6-Minute Walk Test (6MWT), which assessed the cardiorespiratory capacity of the participants, was administered separately from the other tests in a covered and ventilated multi-sport court marked with 30-m lanes. Upon the command (“Go”), the participant walked as fast as possible for 6 min, with only one attempt performed. At the end, the distance covered was recorded in meters (Bittner et al., 1993).

##### 2.4.3.6 Blood collection and BDNF measurement

Blood samples were collected after a 12-h overnight fast and 72 h of exercise abstinence. Samples were drawn from the antecubital vein (4 mL) into vacuum blood collection tubes (Vacutainer®; Becton Dickinson®, Franklin Lakes, United States), centrifuged (3,000 rpm, 10 min at 4 °C), and stored at −80 °C. Serum BDNF levels were measured using a sandwich enzyme-linked immunosorbent assay (ELISA) with a detection range of 0.066–16 ng/mL (Human BDNF ELISA kit - cat# EH42RB, Thermo Fisher Scientific Company, United States) according to the manufacturer’s instructions.

#### 2.4.4 Statistical analysis

The sample size calculation was performed using the G*Power software (Erdfelder, Faul and Buchner, 1996; Kiel, Germany - version 3.1.9.2) based on the main dependent variables: 1) cognitive function - semantic memory and cognitive status (de Oliveira Silva et al., 2019; Yoon et al., 2017); 2) functional fitness–dynamic balance and cardiorespiratory capacity (de Resende-Neto et al., 2021). Thus, a statistical power of 0.80 and an alpha of 0.05 were considered, in addition to an anticipated sample loss of 20% during the intervention.

The data were tabulated and analyzed using JAMOVI software (version 2.4.11). Descriptive statistics were used to summarize the general characteristics of the study participants. The normality of the data was confirmed using the Shapiro-Wilk test, and homogeneity of variances was verified using Levene’s test.

Repeated measures analysis of variance (ANOVA) was used to verify the differences between the interventions. The Bonferroni *post hoc* test was used to identify where the significance occurred. All tests were two-tailed, and the effect size (ES) was calculated according to the equation proposed by Cohen (1988), as well as the classification of each result (trivial: 0.00-0.19; small: 0.20-0.49; moderate: 0.5-0.79; large: 0.8-1.33; and very large: >1.33). The significance level adopted for all tests was p < 0.05.

### 2.5 Results

The initial sample consisted of 89 older women (mean age = 67.1 ± 4.7 years; BMI = 29.29 ± 4.94 kg/m^2^) at risk for MCI (MoCA: 19.32 ± 3.73 points), independent in activities of daily living (IADL: 20.61 ± 0.86 points), and with a low indicative risk of depression (GDS-15: 2.46 ± 1.39). Of these, 68 completed all stages of the intervention, and the experimental groups showed a 91% adherence rate to the training sessions (Table 3).

**TABLE 3:** Baseline Characteristics of participants in the Functional Training (FT), Aerobic (AT), and Control (CG) groups.

Varibles	FT (n = 28)	AT (n = 22)	CG (n = 18)	p
	M ± SD	M ± SD	M ± SD	
Age (years)	67.5 ± 4.8	66.4 ± 4.6	67.5 ± 4.6	0.680
Body weight (Kg)	68.2 ± 11.3	66.0 ± 14.4	67.9 ± 15.6	0.845
Height (meters)	1.53 ± 6.88	1.50 ± 4.91	1.50 ± 5.05	0.171
BMI (Kg/m^3^)	29.29 ± 4.39	28.71 ± 4.68	29.07 ± 6.13	0.775
MoCA	18.64 ± 3.69	19.95 ± 3.75	19.61 ± 3.79	0.446
IADL	20.52 ± 0.96	20.79 ± 0.53	20.56 ± 0.98	0.428
GDS-15	2.50 ± 1.58	2.52 ± 1.12	2.33 ± 1.45	0.899
Years of education	7.5 ± 3.2	8.6 ± 3.8	7.6 ± 4.7	0.659
Medical History				
Diabetes	13 (46%)	08 (40%)	09 (50%)	0.624
Hypertension	18 (64%)	13 (65%)	13 (72%)	0.738
Dyslipidemia	20 (71%)	12 (60%)	13 (72%)	0.270
Medications				
0	03 (10%)	03 (13%)	00 (00%)	0.180
≤ 3	15 (53%)	12 (54%)	7 (38%)	0.560
> 3	10 (35%)	07 (31%)	11 (61%)	0.151

Note: Values are presented as mean and standard deviation (M ± SD). MoCA, Montreal Cognitive Assessment; GDS15 – Geriatric Depression Scale; IADL, Instrumental Activities of Daily Living; BMI, Body Mass Index. One-way ANOVA.

**FIGURE 3:**
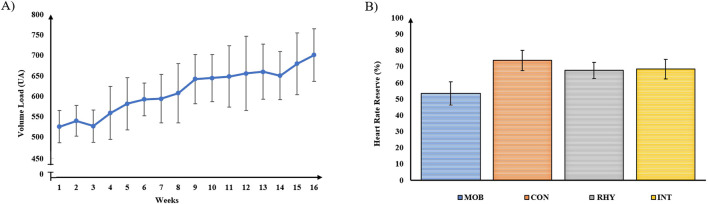
Quantification of training load and monitoring of training intensity. **(A)** Volume load (repetitions x sets x intensity); **(B)** Mean and standard deviation of heart rate reserve. MOB: Mobility; CON: Continuous; RHY: Rhythmic; INT: Interval.

In FT, the volume-load demonstrates that the training load was progressive throughout the 16 weeks (Figure 3A). Conversely, the monitoring of HRres demonstrates that an intensity between 50% and 80% was maintained during the AT sessions (Figure 3B).

**FIGURE 4:**
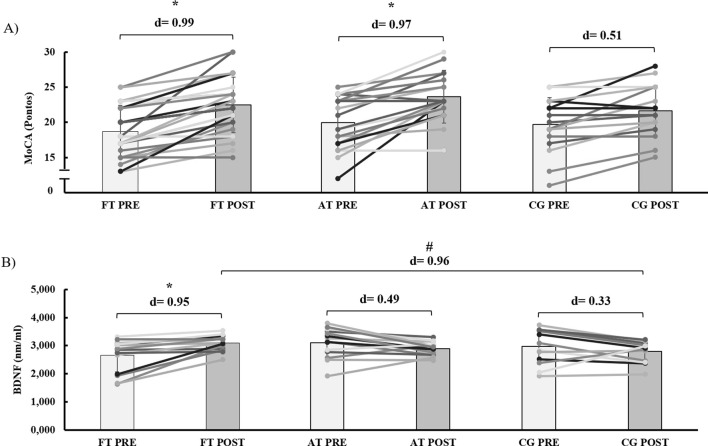
Effects of functional and aerobic training on cognitive status **(A)** and plasma BDNF concentration **(B)** in older women with Mild Cognitive Impairment. FT: Functional Training; AT: Aerobic Training; CG: Control Group.

Following the 16-week intervention period, we observed a significant group*time interaction for cognitive status (MoCA) (F (2, 65) = 3.16; η^2^ = 0.010; p = 0.035), with no significant group effect (F (2, 65) = 0.910; η^2^ = 0.020; p = 0.408) but a significant time effect (F (2, 65) = 89.68; η^2^ = 0.143; p ≤ 0.001). Upon comparing time points, we detected a large effect size increase in both the FT and AT groups compared to baseline values (FT: *d* = 0.99; p ≤ 0.001/AT: *d* = 0.97; p ≤ 0.001). However, the CG showed no significant difference despite a moderate effect size (*d* = 0.51; p > 0.05) (Figure 4A).

Regarding the peripheral quantification of BDNF, we found a significant group*time interaction (F (2, 37) = 9.07; η^2^ = 0.102; p ≤ 0.001), with no significant group effect (F (2, 37) = 0.518; η^2^ = 0.018; p = 0.606) and no significant time effect (F (2, 37) = 0.079; η^2^ = 0.002; p = 0.790). Upon further exploration of the results, we only detected a large effect size increase in the FT group compared to baseline values (*d* = 0.95; p < 0.011). Furthermore, when comparing post-test time points, we observed a difference with a large effect size between the FT and CG groups (*d* = 0.96; p = 0.026) (Figure 4B).

Regarding cognitive function, for which the analyzed variables may influence performance, we observed no significant differences between the groups for short-term memory (DSF), working memory (DSB), semantic memory (FV_QAni), and executive function (FV_QAgru). However, FT showed an improvement with a moderate to large effect size compared to baseline values in semantic memory (*d* = 0.95; p < 0.001) and executive function (*d* = 0.63; p = 0.043). In contrast, AT demonstrated an improvement with a large effect size compared to baseline values only for semantic memory (*d* = 0.97; p < 0.001). The CG showed no adaptations for any of the cognitive variables (Table 4).

**TABLE 4:** Effects of functional and aerobic training on short-term memory, working memory, semantic memory, and executive function in older women with mild cognitive impairment.

Variables *Assessment Time Points*	FT (n = 28)	AT (n = 22)	CG (n = 18)	*p-value* Interaction Group-time	FT vs*.* CG	At vs*.* CG	FT vs*.* AT
Digit Span - Forward (DSF)							
*Baseline (pre)*	16.68 ± 3.97	16.00 ± 3.45	16.06 ± 2.99				
*Post_16_week*	16.50 ± 4.38	17.64 ± 4.11	14.88 ± 3.12	0.009	1.00	1.00	1.00
*Δ% - ES*	−1.08 – 0.05^T^	+10.25–0.43^S^	−7.35 – 0.39^S^				
*CI (95%)*	(15.05–18.15)	(15.05–18.60)	(14.90–18.80)				
Digit Span – Backward (DSB)							
*Baseline (pre)*	6.89 ± 2.48	7.14 ± 3.18	6.89 ± 3.08				
*Post_16_week*	8.00 ± 2.68	8.41 ± 3.36	7.22 ± 2.98	0.424	0.82	1.00	1.00
*Δ% - ES*	+16.11–0.43^S^	+17.79–0.39^S^	+4.78–0.11^T^				
*CI (95%)*	(6.33–8.55)	(6.52–9.02)	(5.67–8.44)				
Verbal Fluency – category animals (number of animals) – VF_Nani							
*Baseline (pre)*	13.64 ± 4.04	14.73 ± 4.70	14.11 ± 4.68				
*Post_16_week*	17.57 ± 4.20*	19.14 ± 4.37*	16.06 ± 4.37	0.173	1.00	1.00	1.00
*Δ% - ES*	+28.81–0.95^L^	+29.94–0.97^L^	+13.82–0.43^S^				
*CI (95%)*	(13.95–17.30)	(15.00–18.85)	(13.90–18.15)				
Verbal Fluency – category animals (number of clusters) – VF_NClus							
*Baseline (pre)*	1,39 ± 1.34	1.68 ± 1.32	1.50 ± 1.34				
*Post_16_week*	2.25 ± 1.40*	2.32 ± 1.46	2.06 ± 1.21	0.822	1.00	1.00	1.00
*Δ% - ES*	+65.25–0.63^M^	+38.10–0.46^S^	+37.33–0.44^S^				
*CI (95%)*	(1.31–2.33)	(1.41–2.57)	(1.13–2.41)				

Note: Values are presented as mean and standard deviation (M ± SD); p ≤ 0.05 (pre/post). Δ%: Percentage change between Baseline (pre) and Post_16. wks: Weeks. ES: Effect Size (^T^Trivial: 0.0-0.19; ^S^Small: 0.2-0.49; ^M^Moderate: 0.5-0.79; ^L^Large: 0.8-1.32; ^VL^Very, Large: >1.33). CI – Confidence Interval.

The * indicates the statistical difference when the p-value ≤ 0.05 in the pre- and post-values. The # indicates the statistical difference between the post-time points.

Regarding cardiorespiratory fitness (6MWT), we observed differences between the groups (FT/CG: p = 0.008; AT/CG: p = 0.041). When compared to baseline, we found an increase in the distance covered for both the FT and AT groups with a moderate effect size (FT: *d* = 0.66; p = 0.002/AT: *d* = 0.56; p = 0.036). In the CG, we observed a non-significant reduction with a small effect size (*d* = 0.35; p = 0.464). Regarding upper limb strength (ULS), we observed no significant differences between the groups. However, when considering changes from baseline, only the FT group showed an increase in handgrip strength in kilogram-force with a moderate effect size (*d* = 0.64; p ≤ 0.001) (Table 5).

**TABLE 5:** Effects of Functional and Aerobic Training on Functional Fitness for activities of daily living in older women with mild cognitive impairment.

Variables *Assessment Time Points*	FT (n = 28)	AT (n = 22)	CG (n = 18)	*p-value* Interaction Group-time	FT vs*.* CG	At vs*.* CG	FT vs*.* AT
Six-Minute Walk Test - 6MWT (meters)							
*Baseline (pre)*	481.27 ± 61.56	479.43 ± 73.31	474.94 ± 78.16				
*Post_16_week*	525.37 ± 71.77*	517.05 ± 58.98*	445.87 ± 85.58	0.001	0.008	0.041	1.00
*Δ% - ES*	+9.16–0.66^M^	+7.85–0.56^M^	−6.12 – 0.35^S^				
*CI (95%)*	(476.00 – 530.50)	(468.00–528.50)	(427.00–494.00)				
Five Times Sit-to-Stand Test – FTSST (seconds)							
*Baseline (pre)*	8.26 ± 1.97	8.00 ± 1.71	7.91 ± 2.37				
*Post_16_week*	7.12 ± 1.77*	7.33 ± 1.55*	8.14 ± 2.26	0.001	1.00	1.00	1.00
*Δ% - ES*	+13.80–0.61^M^	+8.37–0.41^S^	−8.14 – 0.10^T^				
*CI (95%)*	(6.97–8.42)	(6.85–8.49)	(7.12–8.93)				
Floor Rise Test – FRT (seconds)							
*Baseline (pré)*	3.81 ± 0.96	3.91 ± 1.00	3.77 ± 0.86				
*Pós_16_sem*	3.03 ± 0.60*	3.33 ± 0.97*	4.04 ± 0.97	0.001	0.003	0.161	1.00
*Δ% - ES*	+20.47–0.97^L^	+14.83–0.59^M^	−7.16 – 0.29^S^				
*CI (95%)*	(3.08–3.76)	(3.23–4.01)	(3.48–4.33)				
Timed Up and Go – TUG (seconds)							
*Baseline (pre)*	7.57 ± 1.00	7.36 ± 0.72	7.52 ± 0.91				
*Post_16_week*	6.92 ± 0.88*	6.95 ± 0.80*	7.68 ± 1.07	0.001	0.114	0.210	1.00
*Δ% - ES*	+8.58–0.69^M^	+5.57–0.54^M^	−2.12 – 0.16^T^				
*CI (95%)*	(6.91–7.59)	(6.77–7.54)	(7.18–8.03)				
10-m Walk Test - 10MWT (seconds)							
*Baseline (pre)*	5.17 ± 0.64	5.21 ± 0.62	5.32 ± 0.64				
*Post_16_week*	4.85 ± 0.50*	4.85 ± 0.44*	5.23 ± 0.54	0.093	0.079	0.232	1.00
*Δ% - ES*	+6.19–0.56^M^	+6.91–0.67^M^	+1.69–0.15^T^				
*CI (95%)*	(4.78–5.22)	(4.80–5.27)	(5.01–5.54)				
Gallon Jug Shelf Transfer – GJST (seconds)							
*Baseline (pre)*	10.34 ± 1.06	10.56 ± 0.97	10.87 ± 1.29				
*Post_16_week*	9.74 ± 1.00*	10.06 ± 1.01	10.97 ± 1.34	0.018	0.007	0.176	1.00
*Δ% - ES*	+5.80–0.58^M^	+4.73–0.51^M^	−0.91 – 0.04^T^				
*CI (95%)*	(9.60–10.50)	(9.81–10.80)	(10.36–11.50)				
Dressing on and taking off a t-shirt – DTTS (seconds)							
*Baseline (pre)*	13.65 ± 2.72	13.21 ± 2.39	13.31 ± 1.99				
*Post_16_week*	12.00 ± 2.03*	12.64 ± 2.59	13.19 ± 1.97	0.003	1.00	1.00	1.00
*Δ% - ES*	+12.09–0.69 ^M^	+4.31–0.23 ^S^	+0.90–0.06 ^T^				
*CI (95%)*	(11.95–13.70)	(11.95–13.95)	(12.20–14.35)				
Handgrip Strength – HGS (kilogram)							
*Baseline (pre)*	22.20 ± 3.93	21.79 ± 4.67	22.76 ± 4.41				
*Post_16_week*	24.81 ± 4.21*	22.66 ± 3.98	22.52 ± 4.21	0.001	1.00	1.00	1.00
*Δ% - ES*	+11.75–0.64^M^	+3.99–0.20^S^	−1.05 – 0.05^T^				
*CI (95%)*	(22.05–25.20)	(20.45–24.00)	(20.65–24.65)				

Note: Values are presented as mean and standard deviation (M ± SD); p ≤ 0.05 (pre/post). Δ%: Percentage change between Baseline (pre) and Post_16 (% change = 100×final−initial∣initial∣% change = 100×∣initial∣final−initial). wks: Weeks. +: performance improvement. -: performance reduction. ES: Effect Size (^T^Trivial: 0.0-0.19; ^S^Small: 0.2-0.49; ^M^Moderate: 0.5-0.79; ^L^Large: 0.8-1.32; ^VL^Very, Large: >1.33). CI – Confidence Interval.

The * indicates the statistical difference when the p-value ≤ 0.05 in the pre- and post-values. The # indicates the statistical difference between the post-time points.

For the tests assessing functional fitness involving the stand up ability to rise from a chair (FTSST) and gait speed (GS), we observed a difference between the groups only for GS (FT/CG: *d* = 1.25; p = 0.003). When compared to baseline values, both intervention groups improved the time to complete the task in the FTSST (FT: *d* = 0.61; p ≤ 0.001/AT: *d* = 0.41; p = 0.021) and GS (FT: *d* = 0.97; p ≤ 0.001/AT: *d* = 0.59; p ≤ 0.001), with effect sizes ranging from small to large. The CG showed no adaptations following the intervention (Table 5).

Consistently, regarding walking ability, TUG and 10MWT, we observed no significant differences between the groups. When compared to baseline values, both intervention groups reduced the time to complete the task in the TUG (FT: *d* = 0.69; p ≤ 0.001/AT: *d* = 0.54; p = 0.022) and similarly for the 10MWT (FT: *d* = 0.56; p ≤ 0.001/AT: *d* = 0.67; p = 0.011), with moderate effect sizes. The CG showed no adaptations following the intervention (Table 5).

Finally, regarding the tests related to dexterity, GJST and DTTS, we observed differences between the groups only for GJST (FT/CG: *d* = 1.04; p = 0.007) with a large effect size. When compared to baseline values, only the FT group reduced the time for task completion in the GJST (*d* = 0.58; p = 0.004) and similarly for the DTTS (*d* = 0.69; p ≤ 0.001), both with a moderate effect size. The AT and CG showed no adaptations following the intervention (Table 5).

### 2.6 Discussion

The present study aimed to evaluate the effects of 16 weeks of FT and AT on cognitive function, functional fitness, and serum BDNF levels in older women with mild cognitive impairment. Our main finding was that both training protocols promoted consistent improvements in cognitive state and semantic memory. However, only FT improved executive function and increased peripheral BDNF concentration. Regarding functional fitness, we observed larger effect sizes in the ability to stand up and in tasks involving manual dexterity, as well as specifically in handgrip strength, thus confirming our initial hypothesis.

The multisystem adaptations resulting from the combination of multicomponent stimuli with movement specificity for daily activities, together with the individualization and progression of the training load, have been previously evidenced by our group (Pantoja-Cardoso et al., 2023; Vasconcelos et al., 2022; Carrasco-Poyatos et al., 2019). The present study provides a consistent investigation into the effects of physical training on cognitive function and quantification of the training load, which suggests the absence of adaptive stagnation throughout the intervention.

Regarding cognitive status, significant effects with a large effect size (FT: d = 0.99; AT: d = 0.97) were observed for both training protocols. These findings likely stem from the positive adaptation of cardiorespiratory capacity, which is directly linked to enhanced cerebral vascularization (Ferrer-Uris et al., 2022; Dupuy et al., 2015; Tsai et al., 2014). This adaptation promotes increased connectivity between brain regions (Holzschneider et al., 2012), enhancing learning (Ferrer-Uris et al., 2022) and attenuate cognitive decline (Hoffmann et al., 2016). Our results are consistent with previous studies in older adults with MCI (Vega-Ávila et al., 2022; Yong et al., 2021). Furthermore, improvements caused by the FT protocol may be related to visuospatial stimuli (Lee et al., 2024) and the motor complexity of the exercises (Fontes et al., 2020). These stimuli may positively favor the availability of oxygen and nutrients to neural cells (Crichton et al., 2014), with continuous activation of areas related to movement planning and execution (Lee et al., 2024).

Executive function is crucial for problem-solving and the planning of cognitive or motor tasks. It comprises working memory, cognitive flexibility, and inhibitory control, all of which independently contribute to executive function (Diamond, 2013). Only the FT protocol showed significant effects on executive function with a moderate effect size (FT: d = 0.63), which can be attributed to the motor demands of the neuromuscular-dominant block, combined with the high volume of multi-joint and multi-planar exercises. This requires more planning when compared to the rhythmic movements used in the AT protocol with a small effect size (AT: d = 0.46) (La Scala Teixeira et al., 2019). Consequently, greater activation of areas responsible for motor planning, such as the supplementary motor area, premotor cortex, cerebellum, and basal ganglia, is expected (Vints et al., 2024; Kušleikien et al., 2025; Barha et al., 2022).

Specifically, regarding short-term memory (DSF) (FT: d = 0.05; AT: d = 0.43) and working memory (DSB) (FT: d = 0.43; AT: d = 0.39), no significant adaptations were observed for any of the groups, with effect sizes ranging from trivial to small. The ineffectiveness of both FT and AT may stem from the absence of specific short-term memory stimuli (2–10 min) for subsequent recall and manipulation. Possibly, the addition of motor-cognitive dual tasks could provide effective adaptations for working memory (Norouzi et al., 2019). This is because the inclusion of dual tasks will generate greater activation of the prefrontal cortex, which is related to improved short-term and working memory performance (Marvel et al., 2019). Still, some studies that did not use dual tasks found effects with longer intervention durations, as well as the short duration of the current intervention, given that these effects have been observed in studies lasting 24 weeks (Spruill, 2010; Zhidong et al., 2021).

In terms of semantic memory, both groups showed substantial improvement. These adaptations may be attributed to the moderate to high intensity of the sessions, which suggests an increase in cortical vascularization (O’Callaghan et al., 2020), followed by greater excitability of prefrontal and temporal regions (Nocera et al., 2017) particularly the hippocampus (Rivas-Campo et al., 2023), contributing to the reorganization of neural circuits (Smith et al., 2013). Both findings are consistent with the existing literature, demonstrating that multicomponent and aerobic stimuli can promote adaptations in semantic memory (Rivas-Campo et al., 2023).

Pertaining to peripheral BDNF concentration, only the FT protocol showed significant increases compared to both baseline values, with a large effect size (d = 0.95) and the CG (d = 0.96). BDNF is essential for neural plasticity and is highly expressed in brain areas involved in cognitive processing. BDNF improves memory storage and dendritic spine plasticity. The action of BDNF is primarily mediated by the tyrosine kinase receptor B (TrkB) and initiates signaling pathways that promote neuroprotection (Wang et al., 2020; Zhao et al., 2022). Cognitive function and BDNF are modulated by the combination of cardiorespiratory, neuromuscular, and motor stimuli (Venegas-Sanabria et al., 2022; Yan et al., 2023).

Furthermore, at the peripheral level, maintaining the motor complexity in multi-joint and multi-planar exercises likely exerts greater neuromuscular activation, improving stability between the neuromuscular junction and the motor endplate (Sepulveda-Loyola et al., 2025). These alterations may favor an increase in BDNF synthesis within skeletal muscle and its subsequent release into the bloodstream (Imaizumi et al., 2025).

It has been demonstrated that aerobic exercise significantly increases serum BDNF levels, and a significant relationship between BDNF levels and physical activity levels has been reported (Coelho et al., 2014). The lack of response in AT may be related to the maintenance of the training load throughout the intervention period, as progressively applied intensity in aerobic exercise influences the modulation of BDNF concentration (Huang et al., 2024; Babaei et al., 2013). Additionally, genetic factors that can negatively impact BDNF synthesis, such as the Val66Met gene polymorphism and APOEε4 (ε4), were not identified in the groups (Enette et al., 2020; Lemos et al., 2016; Allard et al., 2017). This may have affected the synthesis and increased peripheral BDNF concentration in AT.

For cardiorespiratory fitness, both groups showed moderate improvement (FT: d = 0.66; AT: d = 0.56). In fact, it is widely known that AT applied at moderate to high intensity promotes cardiovascular and respiratory adaptations that favor the enhancement of this physical capacity (Alemayehu and Teferi, 2023). While in FT, the use of sprints, changes of direction, and other movements performed at high intensity (de Resende-Neto et al., 2021; Rivas-Campo et al., 2023), combined with the circuit exercise structure (Gbiri and Amusa, 2020), may lead to positive changes in central and peripheral mechanisms that favor oxygen transport and utilization.

Concerning the ability to stand up, assessed by the FRT (FT: d = 0.97; AT: d = 0.59) and FTSST tests (FT: d = 0.61; AT: d = 0.41), both the AT and FT protocols showed significant improvements with effect sizes ranging from small to large. The rhythmic and high-intensity interval part applied in AT may stimulate muscle power and motor coordination, thus leading to improvements in this ability (Li et al., 2024). In addition, FT is predominantly neuromuscular, with training session parts focused on stimulating power and muscle strength of the lower limbs, in addition to including movements with a motor pattern similar to the functional tests analyzed (Vasconcelos et al., 2020; Carrasco-Poyatos et al., 2019).

As for walking ability, mimicked by the TUG (FT: d = 0.69; AT: d = 0.54) and 10MWT (FT: d = 0.56; AT: d = 0.67) tests, both the AT and FT protocols showed significant improvements with moderate effect sizes. The stimuli targeting velocity and dynamic balance during acceleration and deceleration actions, present in both protocols, stimulate muscle power (Li et al., 2024; Carrasco-Poyatos et al., 2019). It is already known that there is a strong correlation between lower limb power and dynamic balance, as well as usual gait speed (Yee et al., 2021). These stimuli constantly perturb the postural control system and activate stabilizing muscles, promoting adaptations in dynamic balance (Shahtahmassebi et al., 2019).

With respect dexterity, which was analyzed using the GJST (FT: d = 0.58; AT: d = 0.51) and DTTS (FT: d = 0.69; AT: d = 0.23) tests, only the FT protocol showed a significant improvement with moderate effect sizes for both tests. The absence of significant adaptation in the AT group may be due to the lack of task-specific exercises involving the upper limbs. FT presents a result similar to other studies, in which the quantity of exercises targeting the upper limbs, applied with pushing and pulling patterns, may favor dexterity (Aragão-Santos et al., 2024). Other studies involving strength training have highlighted that performing movements with large amplitudes favors gains in joint mobility (Carrasco-Poyatos et al., 2019; DE Matos et al., 2017).

Finally, regarding Handgrip Strength, which is considered a strong predictor of mortality and disability, a significant improvement was observed only for FT (d = 0.64) with moderate effect sizes (Taekema et al., 2010). These adaptations in FT may be attributed to the manipulation of equipment (kettlebell, Bulgarian Bag, Weight Plates) during the strength exercises. This result is consistent with the current literature (Aragão-Santos et al., 2019).

Our findings provide important contributions regarding the effects of different interventions on functional fitness, with particularities not yet evidenced in the scientific community. One limitation of this study is the lack of intensity progression in AT, which may have influenced the absence of significant changes in BDNF and could potentially yield greater effects on functional fitness. However, the percentage of HRR was monitored to maintain moderate to high intensity during the sessions, as suggested in other studies to promote cardiorespiratory adaptations that are associated with improved cognitive function. Another limitation is that the ICC was not calculated; however, according to similar studies of our research group, the tests used exhibit sensitivity above 75% and an ICC greater than 0.80. Finally, the absence of intention-to-treat (ITT) analysis is considered a limitation of this study.

Based on our findings, future research should consider other cognitive subdomains, utilizing more sensitive and specific tests, and also verify the activation of different brain areas during interventions or assessments. We recommend comparisons between men and women, with and without mild cognitive impairment, regarding cognitive and physical performance, verifying the impact of the interventions on quality of life. Furthermore, it is essential to investigate the integration of cognitive and motor stimuli over time, analyzing the residual effect of physical exercise on cognition.

### 2.7 Conclusion

Sixteen weeks of FT and AT promoted improvements in cognitive status and semantic memory, but only FT enhanced overall executive function and BDNF serum levels. Regarding functional fitness, both training modalities improved cardiorespiratory fitness, as well as the ability to rise and walking ability. However, only FT positively altered dexterity and handgrip strength in older women with mild cognitive impairment. These results highlight the importance of structured, targeted training programs that are low-cost and offer several benefits for this specific population, making both protocols feasible for implementation in community or clinical settings that require physical training programs to enhance the autonomy and quality of life of the aging population. Thus, this study contributes evidence supporting non-pharmacological interventions aimed at mitigating cognitive and functional decline in older adults, specifically in women, who are disproportionately affected by MCI.

#### 2.7.1 Permission to reuse and copyright

Upon acceptance for publication, the Frontiers in Physiology requires a Copyright Transfer from all authors. Thus, we, the authors, will confirm through the authorship email sent by the journal that we are aware of the manuscript entitled “Functional Training Improves Cognitive Function, Functional Fitness, And Bdnf Levels In Older Women With Mild Cognitive Impairment: A Randomized Controlled Trial” and we hereby transfer to the Frontiers in Physiology the rights to publication, reproduction, transmission, and distribution of the manuscript, as well as the information contained therein, in its entirety or in part, in printed or electronic versions of the Frontiers in Physiology or in new media developed in the future. Furthermore, the authors permit the creation of versions in other languages of the aforementioned manuscript. The authors are prohibited from publishing the Manuscript without prior approval from the Frontiers in Physiology, in any printed or digital media, with the exception of personal websites. However, the content published herein may be freely used by the authors in the preparation of presentations such as classes, lectures, courses, or conferences.

## Data Availability

The original contributions presented in the study are included in the article/supplementary material, further inquiries can be directed to the corresponding author.
